# Cognition and health-related quality of life in long-term survivors of high-grade glioma: an interactive perspective from patient and caregiver

**DOI:** 10.1007/s00701-024-06037-7

**Published:** 2024-04-03

**Authors:** Jochem K. H. Spoor, Marike Donders-Kamphuis, Wencke S. Veenstra, Sarah A. van Dijk, Clemens M. F. Dirven, Peter A. E. Sillevis Smitt, Martin J. van den Bent, Sieger Leenstra, Djaina D. Satoer

**Affiliations:** 1 Department of Neurosurgery, Erasmus MC – University Medical Center Rotterdam, Doctor Molewaterplein 40, 3015 GD Rotterdam, The Netherlands; 2 HMC, Department of Neurosurgery, The Hague, The Netherlands; 3 Department of Rehabilitation Medicine, Center for Rehabilitation - University of Groningen, University Medical Center Groningen, Groningen, The Netherlands; 4 Department of Neurology, Erasmus MC – University Medical Center Rotterdam, Rotterdam, The Netherlands

**Keywords:** High-grade glioma, Long-term survival, Cognition, Quality of life, Caregivers

## Abstract

**Background:**

The health-related quality of life (HRQoL) and cognition are important indicators for the quality of survival in patients with high-grade glioma (HGG). However, data on long-term survivors and their caregivers are scarce. We aim to investigate the interaction between cognition and HRQoL in long-term survivors, their caregivers’ evaluations, and the effect on caregiver strain and burden.

**Methods:**

21 long-term HGG (8 WHO grade III and 13 WHO grade IV) survivors (survival ≥ 5 years) and 15 caregivers were included. Cognition (verbal memory, attention, executive functioning, and language), HRQoL, anxiety and depression, caregiver strain, and caregiver burden were assessed with standardized measures. Questionnaires were completed by patients and/or their caregivers.

**Results:**

Mean survival was 12 years (grade III) and 8 years (grade IV). Cognition was significantly impaired with a large individual variety. Patients’ general HRQoL was not impaired but all functioning scales were deviant. Patient-proxy agreement was found in most HRQoL subscales. Three patients (14%) showed indications of anxiety or depression. One-third of the caregivers reported a high caregiver strain or a high burden. Test scores for attention, executive functioning, language, and/or verbal memory were correlated with perceived global health status, cognitive functioning, and/or communication deficits. Caregiver burden was not related to cognitive deficits.

**Conclusions:**

In long-term HGG survivors maintained HRQoL seems possible even when cognition is impaired in a large variety at the individual level. A tailored approach is therefore recommended to investigate the cognitive impairments and HRQoL in patients and the need for patient and caregiver support.

## Introduction

Despite intensive combination treatment with surgery, radiotherapy, and chemotherapy, high-grade gliomas (HGG) still have a poor prognosis. The tumor itself, the tumor treatments, co-morbid conditions such as epilepsy, and medication all may impair brain function, resulting in impaired cognition [7, 20]. This becomes more relevant over time, thereby affecting long-term survivors substantially more than short-term survivors [9]. Prolonged survival is less meaningful if cognition and well-being are not preserved [4]. In addition, cognitive functioning and health-related quality of life (HRQoL) are positively correlated with survival [18, 24, 41]. However, data on long-term HGG survivors is limited.

Cognition can be measured by validated tests and by questionnaires. In brain tumor patients, results on cognitive tests were not always in accordance with perceived cognitive functioning [12, 40]. In addition, it is common that perceived cognition differs when rated by the patient or caregiver [6, 13, 40]. Knowing how to assess cognition optimally is essential to referring for support or rehabilitation adequately.

Without a doubt, the diagnosis of an HGG impacts both the patient and the caregiver. Caring for an HGG patient brings psychological distress and a heavy burden [1, 17, 30, 31]. For caregivers of HGG long-term survivors, this situation lasts for years. How a patients ‘ cognitive function influences the caregiver’s strain and burden is unknown.

In this study, we primarily aim to investigate the cognitive status of long-term HGG survivors. Various tests that measure different cognitive functions and questionnaires on HRQoL and well-being (filled out by the patient and caregiver) are administered. Secondarily, we focus on the influence of cognitive functioning on the perceived HRQoL of the patient and the caregiver’s strain and burden.

## Methods and materials

This study was conducted between January 2019 and July 2020 after screening a departmental database to select adult patients with an initial diagnosis of glioma WHO grade III or IV. All patients were treated with surgery and combinations of radiotherapy and chemotherapy between 1999 and 2014 and survived at least five years after diagnosis. If deemed feasible a maximal safe resection was performed under awake conditions or under general anesthesia. All other patients underwent a navigation based biopsy. Because the data was collected prior to 2021, the 2016 WHO tumor classification was used [22]. Patients with stable diseases and their caregivers were included. Patients who were unable to perform the tests or who were not native speakers of the Dutch language were excluded. A cohort of 36 patients was identified from our departmental database. Fifteen patients were excluded because of tumor progression (*n *= 4), decease (*n* = 3), refusal (*n* = 3), relocation to another region (*n* = 2), participation in a different study (*n* = 1), a different tumor (*n* = 1), or another mother tongue (= 1). Sociodemographic and clinical characteristics were collected. The Ethical Committee of Erasmus MC Rotterdam approved the study (MEC 2017–1152). All participants gave written informed consent.

Table 1 shows the demographic and clinical characteristics of the 21 included patients and 15 caregivers. The tumor was localized in the left hemisphere in 8 patients (38%), the right hemisphere in 12 patients (57%), and multifocal in one patient (5%). Histological analysis showed WHO-grade III (anaplastic astrocytoma/anaplastic oligodendroglioma) in eight patients (38%), and glioblastoma (WHO grade IV) in 13 patients (62%). See Table 1 for further molecular characterization of these tumors. The mean survival at cognitive assessment was 12 years in grade III (range 7–16 years) and eight years in grade IV (range 5–20 years).

**Table 1:** Demographic and clinical characteristics; y = years; * WHO classification 2016 [22]

	Value (%)
Sex: male/female	12 (57%) /
	9 (43%)
Mean age in years (range)	51 (39–70 y)
Mean years of education (range)	15 (12–20 y)
Handedness: right/left	19 (90%) / 2 (10%)
Tumor location	
Left	8 (38%)
• Frontal	4 (19%)
• Temporal	1 (5%)
• Parieto-occipital	1 (5%)
• Occipital	2 (10%)
Right	12 (57%)
• Frontal	7 (33%)
• Parietal	1 (5%)
• Parieto-occipital	1 (5%)
• Temporoparietal	2 (10%)
• Hippocampal	1 (5%)
Multifocal	1 (5%)
Histology*	
WHO-grade III	8 (38%)
• Anaplastic astrocytoma	5 (24%)
• IDH mutant, MGMT methylated	2 (10%)
• Not specified	3 (14%)
• Anaplastic oligodendroglioma	3 (14%)
• IDH mutant, MGMT methylated	1 (5%)
• Not specified	2 (10%)
WHO-grade IV	13 (62%)
• Glioblastoma	12 (57%)
• IDH mutant, MGMT methylated	3 (14%)
• IDH mutant, MGMT wildtype	3 (14%)
• IDH wildtype, MGMT methylated	3(14%)
• Not specified	3 (14%)
• Gliosarcoma	1 (5%)
Type of surgery:	
• Resection under general anesthesia	17 (81%)
• Awake resection	1 (5%)
• Biopsy	3 (14%)
Postoperative radiotherapy + temozolomide	21 (100%)
Mean survival in years at neuropsychological evaluation	
• Grade III (range)	12 (7-16y)
• Grade IV (range)	8 (5-20y)
Caregivers (*n* = 15)	
Sex: male/female	6 (40%) / 9 (60%)

Cognitive tests on the domains of verbal memory (Hopkins Verbal Learning Test, HVLT, [3]), attention and executive functioning (Trail Making Test, TMT, [38]), and language (Boston Naming Test, BNT [19]; shortened Token Test, TT, [8]; Diagnostic Instrument for Mild Aphasia, DIMA, [32]; category fluency [23] and letter fluency [33]) were administered. Questionnaires on HRQoL (EORTC QLQ-C30, [39]; EORTC QLQ-BN20, [25]), anxiety and depression (Hospital Anxiety and Depression Scale, HADS, [44]), caregiver strain (Caregiver Strain Index, CSI, [28]), and caregiver burden (Zarit Burden Interview, ZBI, [2]) were filled out by each patient and/or caregiver. Table 2 describes all subtests and questionnaires. Tests were administered by an experienced clinical linguist (DS). Tests and questionnaires were scored according to standardized scoring criteria. Individual patients’ test scores were converted into z-scores using the mean and standard deviation of the matched normative data on that test. A z-score between -1.5 and -2.0 reflects a mild impairment, and a z-score of ≤ -2.0 reflects a severe impairment [21].

**Table 2:** Administered cognitive tests and questionnaires

Cognitive tests	Verbal memory	Verbal learning, immediate and delayed recall and delayed recognition	Hopkins Verbal Learning Test (HVLT): direct recall, delayed recall, recognition true positives, recognition false positives [3]
	Attention and executive functioning	Visuomotor speed, (divided) attention, mental flexibility	Trail Making Test (TMT): A, B, B/A [38]
	Language	Word retrieval	Boston Naming Test (BNT) [19]
		Incidence and severity of aphasia, language comprehension	Shortened Token Test (TT) [8]
		Phonology, semantic judgment + word retrieval, spontaneous speech in context	Diagnostic Instrument for Mild Aphasia (DIMA): repetition, semantic odd-picture out, sentence completion [32]
		Flexibility of semantic and phonological thought	Category fluency: animals, professions [23]
			Letter fluency [33]
Questionnaires	Quality of Life (patient and caregiver)	Quality of life and general cancer symptoms	EORTC QLQ-C30 [39]
		Quality of life and specific brain tumor symptoms	EORTC QLQ-BN20 [25]
	Anxiety and depression (patient)	Anxiety and depression	Hospital Anxiety and Depression Scale (HADS) [44]
	Caregiver strain and burden (caregiver)	Caregiver strain	Caregiver Strain Index (CSI) [28]
		Caregiver burden	Zarit Burden Interview (ZBI) [2]

Statistical analyses were performed with SPSS (version 25). After testing for normal distribution, parametric and/or non-parametric tests were used. A one-sample t-test was used to compare patients to published normative data healthy controls. Statistically deviating test results were used in the following analyses. Independent samples t-tests were used for subgroup analysis on hemispheric location, tumor grade, and survival (under or above 12 years[9]) and to analyze differences in ratings between patients and caregivers. Associations between cognitive tests and HRQoL (patient and caregiver reports) and caregiver burden were analyzed by Pearson correlations. The level of significance was set at *p* < 0.05.

## Results

### Cognitive tests

At group level, test scores in all cognitive domains were significantly (*p* < 0.05) lower in the long-term HGG survivors compared to normative data. Table 3 shows the results and p-values for each subtest. Three out of four subtests for verbal memory differed significantly compared to healthy controls, and all subtests for attention and executive functioning were substantially lower. For language, three out of eight subtests were significantly impaired. Subgroup analyses on hemispheric localization and tumor grade revealed no significant differences in any subtest. Patients with a 12-year or longer survival, performed significantly lower on a verbal memory test (HVLT Delayed recall, *p* = 0.003), but no other subtests differed significantly.

**Table 3:** Results of the cognitive tests on group level by cognitive domain; *n* = Number of patients who completed the test, as some tests were not completed in all patients due to fatigue or paresis (TMT) * = *p* ≤ 0.05, significantly lower compared to healthy controls

Domain	Test	*n*	Subtest	Mean (z-score)
Verbal memory	Hopkins Verbal Learning Test (HVLT) [3]	19	Direct recall	-1.64*
			Delayed recall	-1.63*
			Recognition: true positives	-0.84*
			Recognition: false positives	-0.52
Attention and executive functioning	Trail Making Test (TMT) [38]	18	A	-1.40*
			B	-1.59*
			B/A	-0.98*
Language	Boston Naming Test (BNT) [19]	20		-1.37*
	Shortened Token Test (TT) [8]	20		-0.57
	Verbal Fluency [23, 33]	18	Category: Animals	-1.11*
			Category: Professions	-1.32*
			Letter	-0.43
	Diagnostic Instrument Mild Aphasia (DIMA) [32]	19	Repetition	-1.04
			Semantic out-picture-out	-0.62
			Sentence completion	-0.18

At the individual level, a large variety in individual cognitive performance was found. Fifteen out of 21 patients completed all subtests. Only one patient (5%) showed no cognitive impairments. All other patients were mildly impaired (z ≤ 1.50) on one to five subtests (mean 1.27, SD 1.33) and severely impaired (z ≤ 2.00) on one to eight subtests (mean 3.07, SD 2.49).

### Questionnaires

Patients’ global health status (QLQ-C30) did not differ significantly from normative data (*p* > 0.05). In contrast, all functional scales were substantially lower (see Table 4) than normative data (*p* ≤ 0.05). Survival, hemispheric localization, and tumor grade subgroup analyses revealed no significant differences between groups (*p* > 0.05). Patient-proxy agreement was found in all subscales except emotional functioning (*p* ≤ 0.05). Patients reported a lower level of emotional functioning than their caregivers reported about the patient.

**Table 4:** Subscales EORTC QLQ-C30 and QLQ-BN20 filled in by patient and caregiver; SD = standard deviation; * = *p* ≤ 0.05, significantly lower compared to healthy controls; ** = *p* ≤ 0.05, significant difference between patient and caregiver report. For QLQ-BN20 Communication deficit no normative data are available

	Patient report		Caregiver report	
	Mean	SD	Mean	SD
QLQ-C30 Global health status	75.17	17.00	75.47	18.68
QLQ-C30 Physical functioning	76.23*	25.45	72.53	27.22
QLQ-C30 Role functioning	67.14*	29.29	58.93	32.62
QLQ-C30 Emotional functioning	86.23*	14.36	92.20**	9.76
QLQ-C30 Cognitive functioning	73.29*	21.07	75.47	18.68
QLQ-C30 Social functioning	70.49*	24.56	67.80	28.37
QLQ-BN20 Communication deficit	18.97	21.70	19.93	26.35

Emotional well-being was measured in patients and their caregivers. Three patients had a deviant score on the HADS. Two of them had high levels of symptoms of anxiety, and one had symptoms of depression. Five caregivers reported a high caregiver strain on the CSI. Four caregivers reported a high burden on the ZBI. Subgroup analysis on the sex of the caregiver showed no significant differences.

### Correlations

Table 5 presents the correlations between cognitive test scores and HRQoL-questionnaires. Significant correlations (*p* ≤ 0.05) were found between attention and executive functioning (TMT). In addition, perceived global health status (QLQ-C30) and cognitive functioning reported by both patient and caregiver (QLQ-C30) correlated significantly. Category fluency is correlated with perceived cognitive functioning (QLQ-C30) when reported by the patient (*r* = 0.487) and is associated with communication deficits (QLQ-BN20) reported by both the patient (*r* = -0.540) and caregiver (*r* = -0.596). Verbal memory (HVLT, *r* = -0.697) and word finding (BNT, *r* = -0.565) correlated with communication deficits reported by the caregiver. Caregiver burden (ZBI) is not associated (*p* > 0.05) with any of the cognitive subtests.

**Table 5:** Correlations between cognitive tests and questionnaires. QL2 = Global health status, CF = cognitive functioning, CD = communication deficit, ZBI = Zarit Burden Interview, HVLT = Hopkins Verbal Learning Test, TMT = Trail Making Test, BNT = Boston Naming Test ** = correlation is significant at the 0.01 level (2-tailed), * = correlation is significant at the 0.05 level (2-tailed)

	HRQoL patient report						HRQoL caregiver report						Burden (caregiver)	
	QLQ-C30 QL2		QLQ-C30 CF		QLQ-BN20 CD		QLQ-C30 QL2		QLQ-C30 CF		QLQ-BN20 CD		ZBI	
	r	p	R	P	r	p	r	p	r	p	r	p	r	p
HVLT Direct recall	0.129	.609	0.177	.482	-0.207	.409	0.018	.952	0.018	.952	-0.498	.083	0.056	.856
HVLT Delayed recall	0.275	.286	-0.028	.914	-0.256	.322	0.228	.476	0.228	.476	-0.697^*^	.012*	-0.167	.604
HVLT Recognition: true positives	0.212	.414	0.018	.946	-0.015	.955	0.149	.645	0.149	.645	-0.471	.122	-0.299	.344
TMT A	0.623	.006**	0.622	.006**	-0.345	.160	0.319	.311	0.319	.311	0.134	.677	-0.319	.313
TMT B	0.585	.011*	0.452	.060	-0.290	.244	0.602	.038*	0.602	.038*	-0.228	.476	-0.512	.089
TMT B/A	0.367	.134	0.145	.566	-0.192	.446	0.621	.031*	0.621	.031*	-0.372	.234	-0.505	.094
BNT	-0.071	.773	0.445	.056	-0.391	.098	-0.202	.471	-0.202	.471	-0.565	.028*	0.335	.241
Category fluency: animals	0.156	.551	0.487	.048*	-0.540	.025*	0.443	.129	0.443	.129	-0.596	.032*	-0.363	.246
Category fluency: professions	-0.094	.721	0.067	.797	-0.325	.203	-0.125	.683	-0.125	.683	-0.281	.352	0.150	.641

## Discussion

In this study, we found that the cognitive status of a cohort of 21 long-term HGG survivors was impaired in multiple cognitive domains. Despite this, global health status as measured by QLQ-C30 is intact. Patient-proxy agreement is found on most subscales in HRQoL questionnaires. An elevated caregiver burden was found in some caregivers but was not related to patients’ cognitive status.

For the cognitive tests, we discovered that in almost all patients cognition was impaired in terms of verbal memory, attention, executive functioning and language. This is in line with Habets et al. [14]. Steinbach et al. [36] also reported attention problems in long-term HGG survivors. However, in their sample, verbal memory was preserved. At the individual level, 95% of our patients had mild or severe impairments in at least one subtest. Previous research on long-term HGG survivors found that 38–100% of patients had mild to severe impairments [11, 14, 16, 36]. Differences may be explained by the quality of neurocognitive reports, that is, how cognition was measured (screening or test), by the definitions of the cognitive domains, and by the thresholds of the deviant scores [15].

For the HRQoL-questionnaires, we revealed that global health status was not deviant compared to healthy controls. Some earlier studies in long-term HGG survivors also reported unaffected quality of life [4, 11, 36], whereas others reported lower [14] and higher levels [27] in patients compared to healthy controls. Long-term HGG survivors coping with the side-effects of their treatment may re-evaluate their internal standards of HRQoL, which may explain the perceived good HRQoL [35]. In contrast to global health status, our patients rated reduced functioning on all subscales. In studies using the QLQ-C30, cognitive and social functioning was also significantly lower compared to controls [11, 14] apart from physical and role functioning. Patients in these studies had different diagnoses, which could have accounted for the differences in comparison to our patient group.

Except for emotional functioning, no differences in perceived HRQoL were found between HGG patients and their caregivers in our study. Literature on low-grade glioma describes both low agreement [10, 34] and moderate agreement [10, 13, 40]. Low agreement is explained by cognitive impairments because patients may be unaware of their cognitive deficits in everyday life [10]. Our patients had cognitive impairments, but despite this, no differences were found in most measurements.

There were two patients who showed indications for anxiety and one patient who showed indications of signs of depression. In other studies on long-term survivors, percentages differ from 10–35 [11, 27, 36], however, the numbers of patients are very small. All are of limited sample size. It has been observed that caregivers recognize depression better than patients [5, 29], possibly causing an underscore. Despite these difficulties, measuring the signs of depression remains vital as it is related to shorter survival [24] and is a very relevant factor to quality of life.

Indications of high burden and caregiver strain were reported in one-third of our caregivers, which is in line with caregivers of long-term meningioma survivors [43]. In the literature on caregivers of HGG patients, caregiver burden is only reported shortly after diagnosis, when it is extremely high [26, 31]. In this phase, the influence of patients’ cognition on caregiver burden is unclear. Sterckx et al. [37] describe in their systematic review cognitive deficits as the most significant challenge for caregivers to deal with. However, most of their included studies did not measure caregiver burden with standardized measurements. In our sample, caregiver burden cannot be explained by the patient's cognitive functioning.

Although several studies among HGG patients, such as Wefel et al. [42], have reported on both neurocognitive symptoms and HRQoL, our study is the first to correlate the results of cognitive tests to perceived HRQoL. In meningioma and low-grade glioma the association between perceived executive functioning and the outcome of cognitive tests remains unclear [40]. In our study, attention and executive functioning (TMT) and language (Category fluency) were found to be related to perceived global health status (QLQ-C30), cognitive functioning (QLQ-C30), and communication deficit (QLQ-BN20), indicating that the test used could objectify perceived cognitive functioning and language or communication deficits.

Limitations in our study are due to a small sample size dictated by the scarcity of long-term survival in HGG. Furthermore, not all patients could complete all subtests, and not all their caregivers could be included. The continuation of data collection among long-term survivors and their caregivers is therefore of utmost importance in order to draw more solid conclusions.

Future research, including a baseline examination is needed to assess the agreement in patient and caregiver ratings and to determine which factors influence caregiver burden in caregivers of brain tumor patients in general and in long-term survivors. Furthermore, care and research is to focus both on impairments and on activity limitations and participation restrictions. In this way, the needs for rehabilitation and support for the patient and caregiver can be identified and addressed, with cognitive rehabilitation and family-centered care becoming part of the future standard of care for long-term survivors. Furthermore, future neuro-oncological therapies are to focus on survival as well as cognition, with HRQOL being one of the primary outcome measures.

## Conclusion

Long-term HGG survivors have impaired cognition in multiple cognitive domains at the group level, with a wide range at the individual level. However, global health status is intact despite lower functional scales. Patient-proxy agreement was found in most HRQoL subscales. In long-term HGG survivors, we strongly recommend a patient-proxy tailored approach using both cognitive tests and HRQoL questionnaires to investigate individual cognitive impairments, quality of life, and caregiver strain and burden.

## Data Availability

Data are available at request.

## Abbreviations

BNT: Boston Naming Test
CSI: Caregiver Strain Index
DIMA: Diagnostic Instrument for Mild Aphasia
EORTC: European Organization for Research and Treatment of Cancer
HADS: Hospital Anxiety and Depression Scale
HGG: High-grade glioma
HRQoL: Health-related quality of life
HVLT: Hopkins Verbal Learning Test
TMT: Trail Making Test
TT: Token Test
QLQ: Quality of Life Questionnaire
ZBI: Zarit Burden Interview
